# Supplementary material for: Tissue Distribution and Immunomodulation in Channel Catfish (Ictalurus punctatus) Following Dietary Exposure to Polychlorinated Biphenyl Aroclors and Food Deprivation
Source: Int J Environ Res Public Health. 2020 Feb 14;17(4):1228. doi: 10.3390/ijerph17041228 (PMC7068257; doi:10.3390/ijerph17041228)
Supplement: Supplementary file 1 [file ijerph-17-01228-s001.pdf]

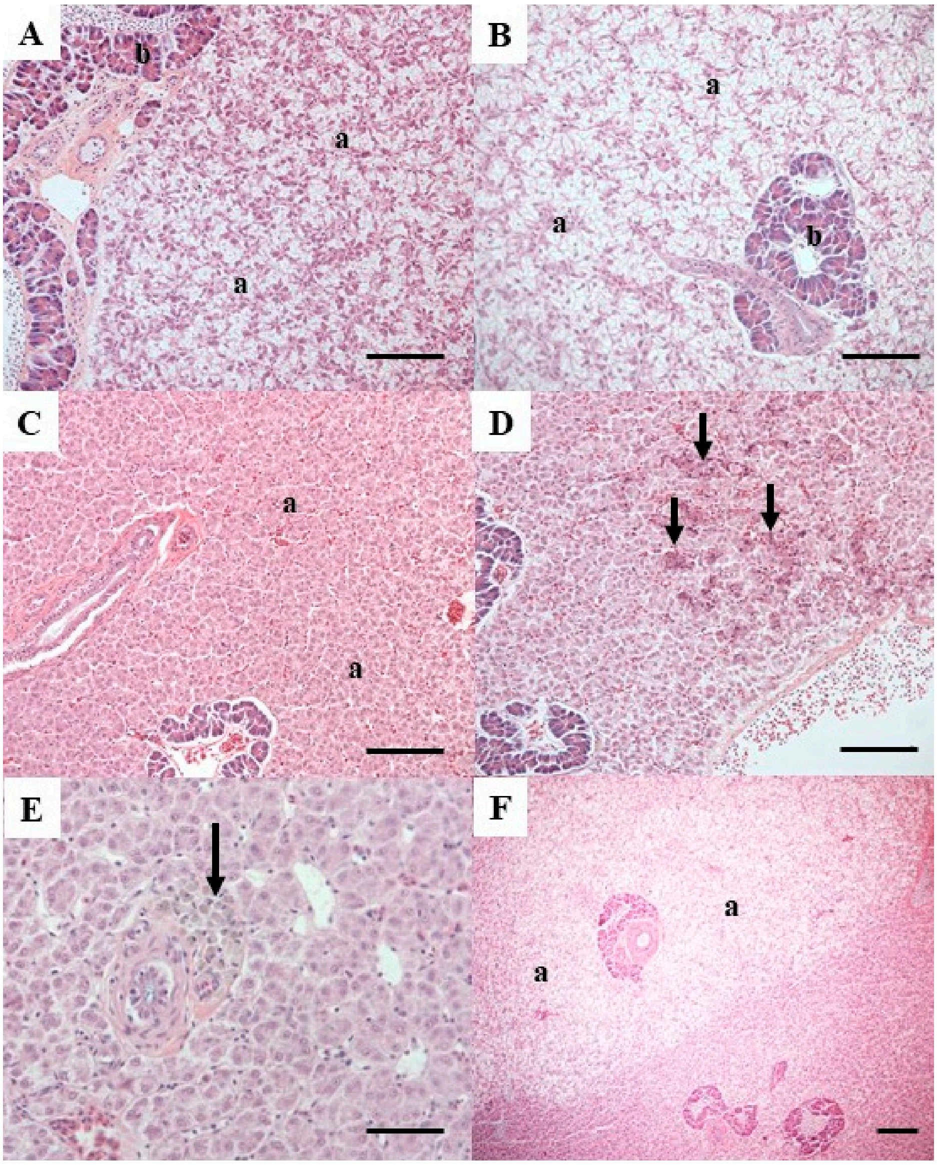

Figure S1: Microscopic observations in liver tissue from control and PCB-exposed channel catfish. A. Liver from control-fed fish with hepatic (a) and pancreatic (b) tissue. Hepatocytes are highly vacuolated. Scale bar equals 100 µm. B. Liver from PCB-fed fish with hepatic (a) and pancreatic (b) tissue. Hepatocytes are highly vacuolated. Scale bar equals 100 µm. C. Liver from fish from the CNF treatment with hepatic (a) and pancreatic (b) tissue. Hepatocytes lack the lipid and glycogen vacuoles of fish that were fed. Scale bar equals 100 µm. D. Liver from fish in the PNF treatment with dark-staining regenerative cells (arrows) throughout the parenchyma. Scale bar equals 100 µm. E. Liver from fish in the PNF treatment with a focal area of cells (arrows) containing yellowish-brown ceroid or lipofuscin pigments. Hepatocytes (a) lack lipid or glycogen vacuoles. Scale bar equals 50 µm. F. Vacuolated preneoplastic foci (a) within the liver of a PNF fish. Scale bar equals 100 µm. Hematoxylin and eosin stain.

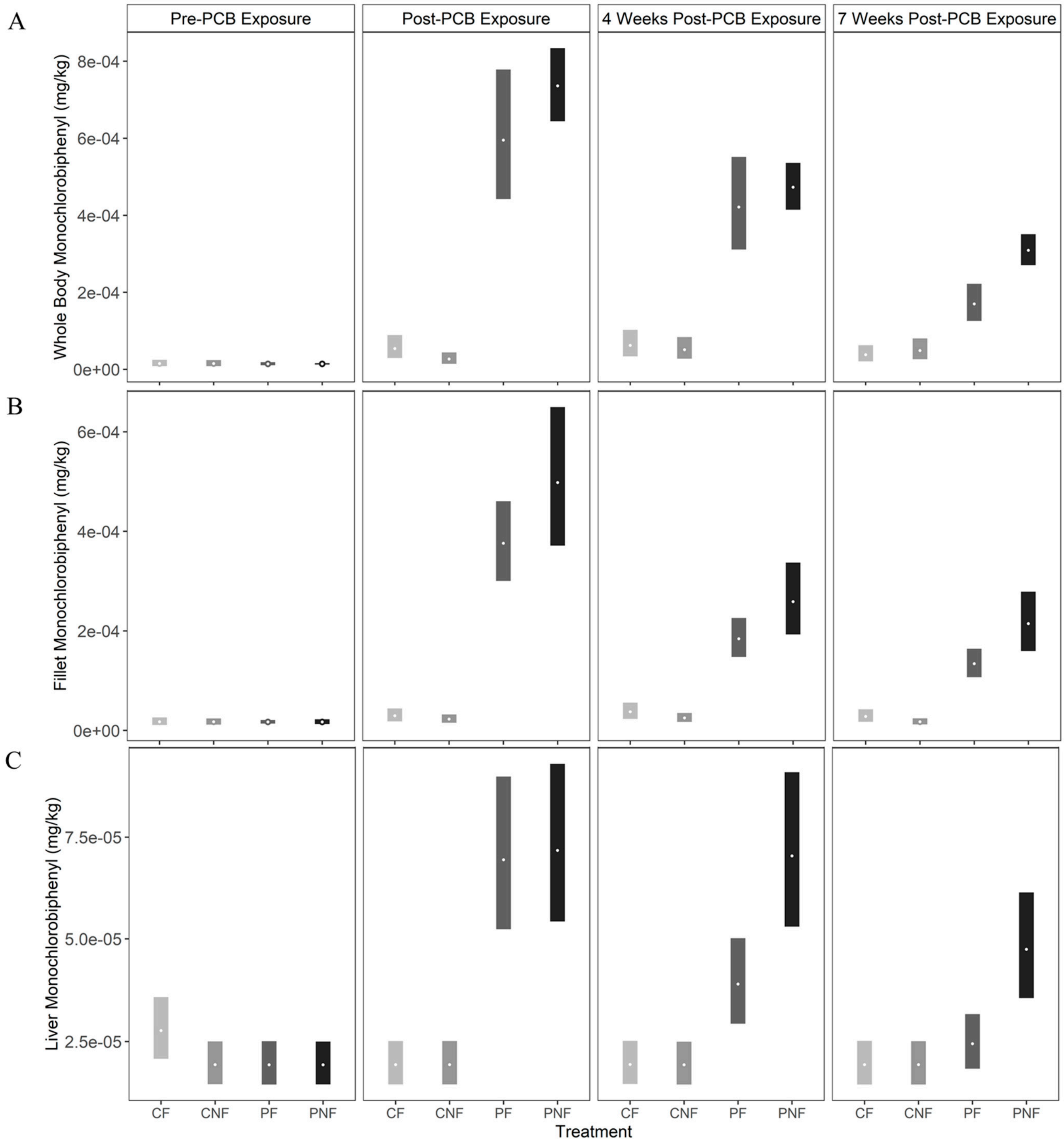

Figure S2: Posterior mean (open circles) and 90% credible intervals (bars) for monochlorobiphenyl concentrations in whole body (A), fillet (B), and liver (C) tissues for channel catfish in each of four treatments (CF = control and fed, CNF = control not fed, PF = PCB exposed and fed, PNF = PCB exposed and not fed). PCB concentrations were measured before PCB exposure, after 11 weeks of PCB exposure, and four and seven weeks after PCB exposure during which fish in the CNF and PNF treatments were also food deprived.

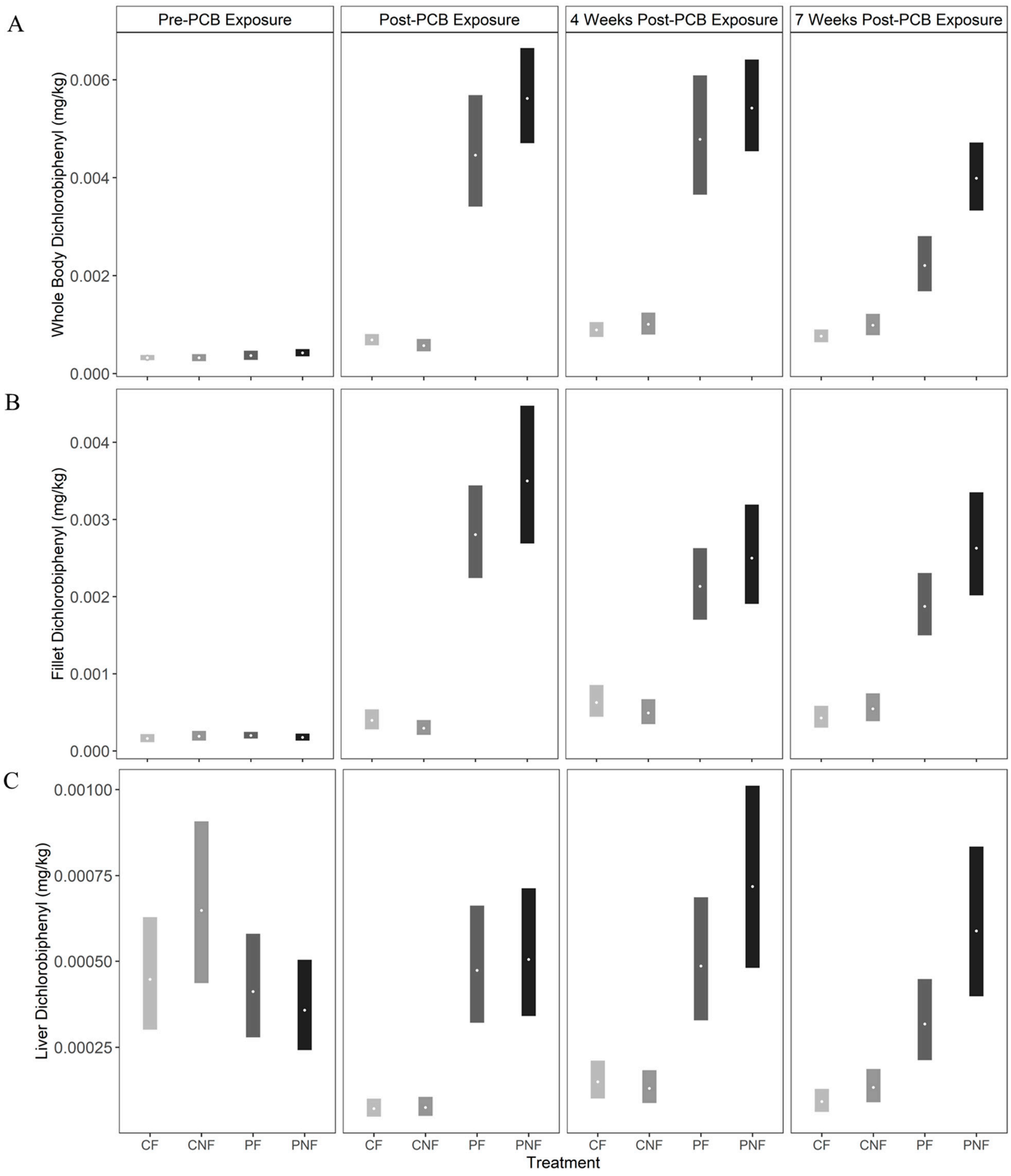

Figure S3: Posterior mean (open circles) and 90% credible intervals (bars) for dichlorobiphenyl concentrations in whole body (A), fillet (B), and liver (C) tissues for channel catfish in each of four treatments (CF = control and fed, CNF = control not fed, PF = PCB exposed and fed, PNF = PCB exposed and not fed). PCB concentrations were measured before PCB exposure, after 11 weeks of PCB exposure, and four and seven weeks after PCB exposure during which fish in the CNF and PNF treatments were also food deprived.

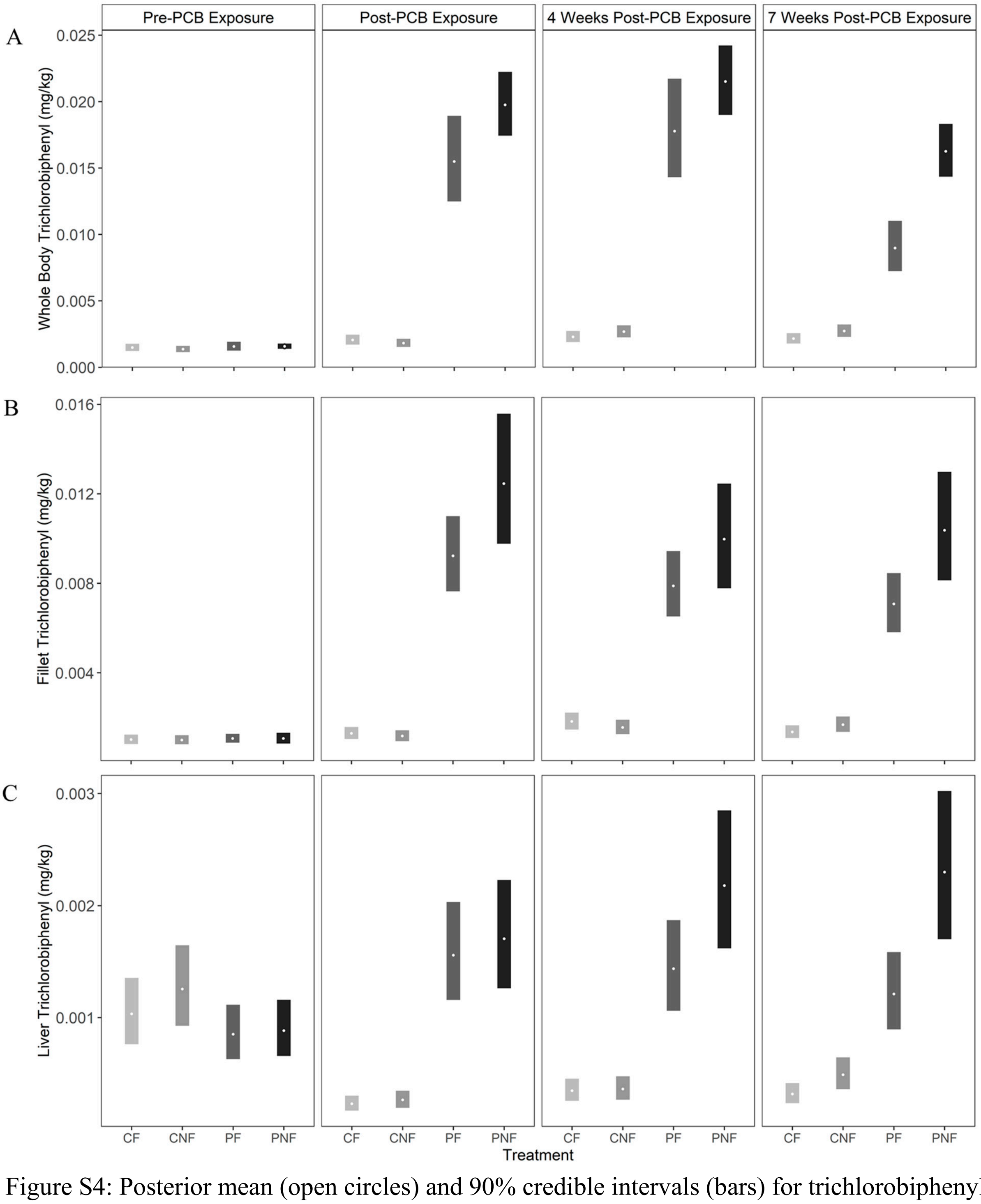

Figure S4: Posterior mean (open circles) and 90% credible intervals (bars) for trichlorobiphenyl concentrations in whole body (A), fillet (B), and liver (C) tissues for channel catfish in each of four treatments (CF = control and fed, CNF = control not fed, PF = PCB exposed and fed, PNF = PCB exposed and not fed). PCB concentrations were measured before PCB exposure, after 11 weeks of PCB exposure, and four and seven weeks after PCB exposure during which fish in the CNF and PNF treatments were also food deprived.

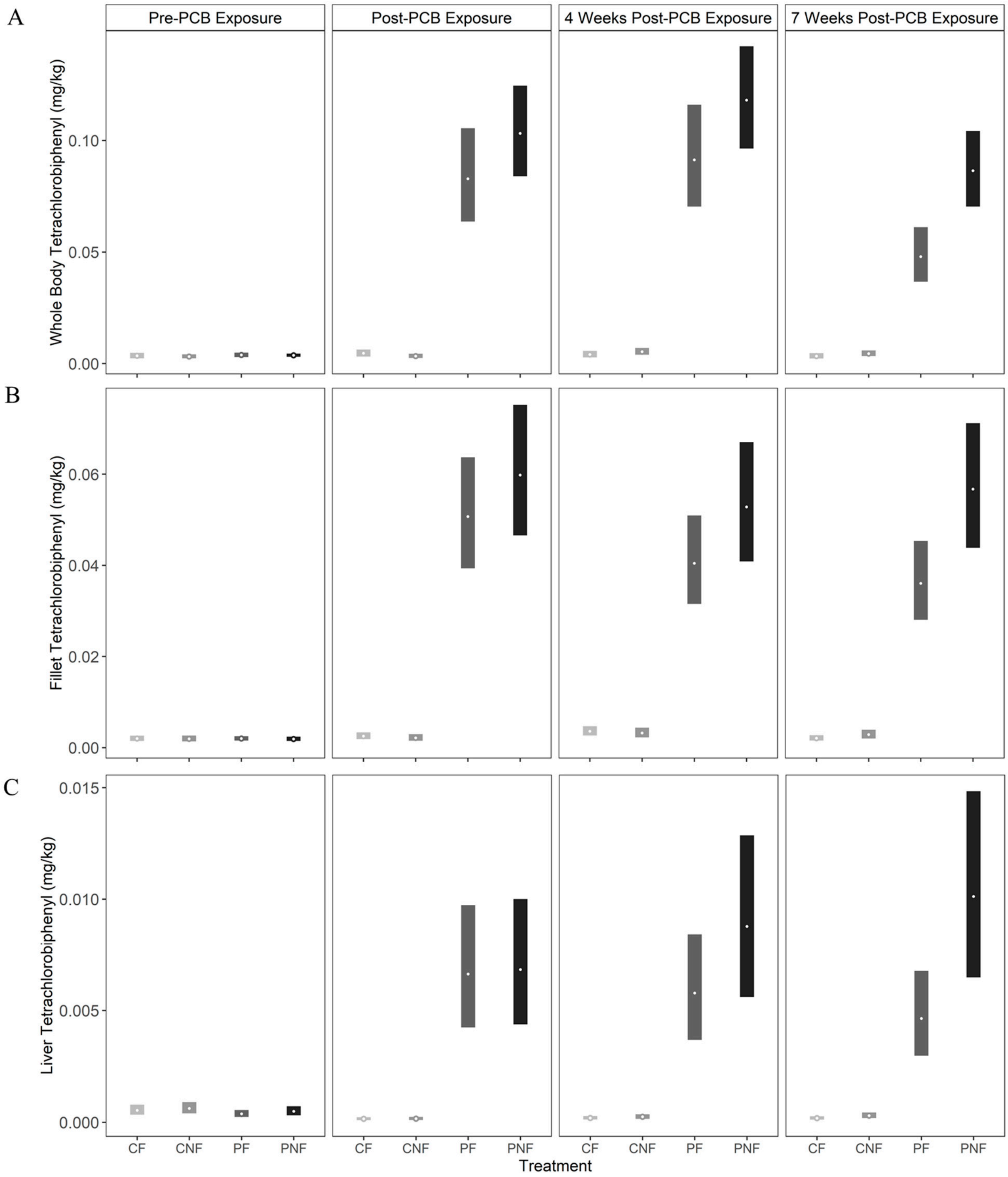

Figure S5: Posterior mean (open circles) and 90% credible intervals (bars) for tetrachlorobiphenyl concentrations in whole body (A), fillet (B), and liver (C) tissues for channel catfish in each of four treatments (CF = control and fed, CNF = control not fed, PF = PCB exposed and fed, PNF = PCB exposed and not fed). PCB concentrations were measured before PCB exposure, after 11 weeks of PCB exposure, and four and seven weeks after PCB exposure during which fish in the CNF and PNF treatments were also food deprived.

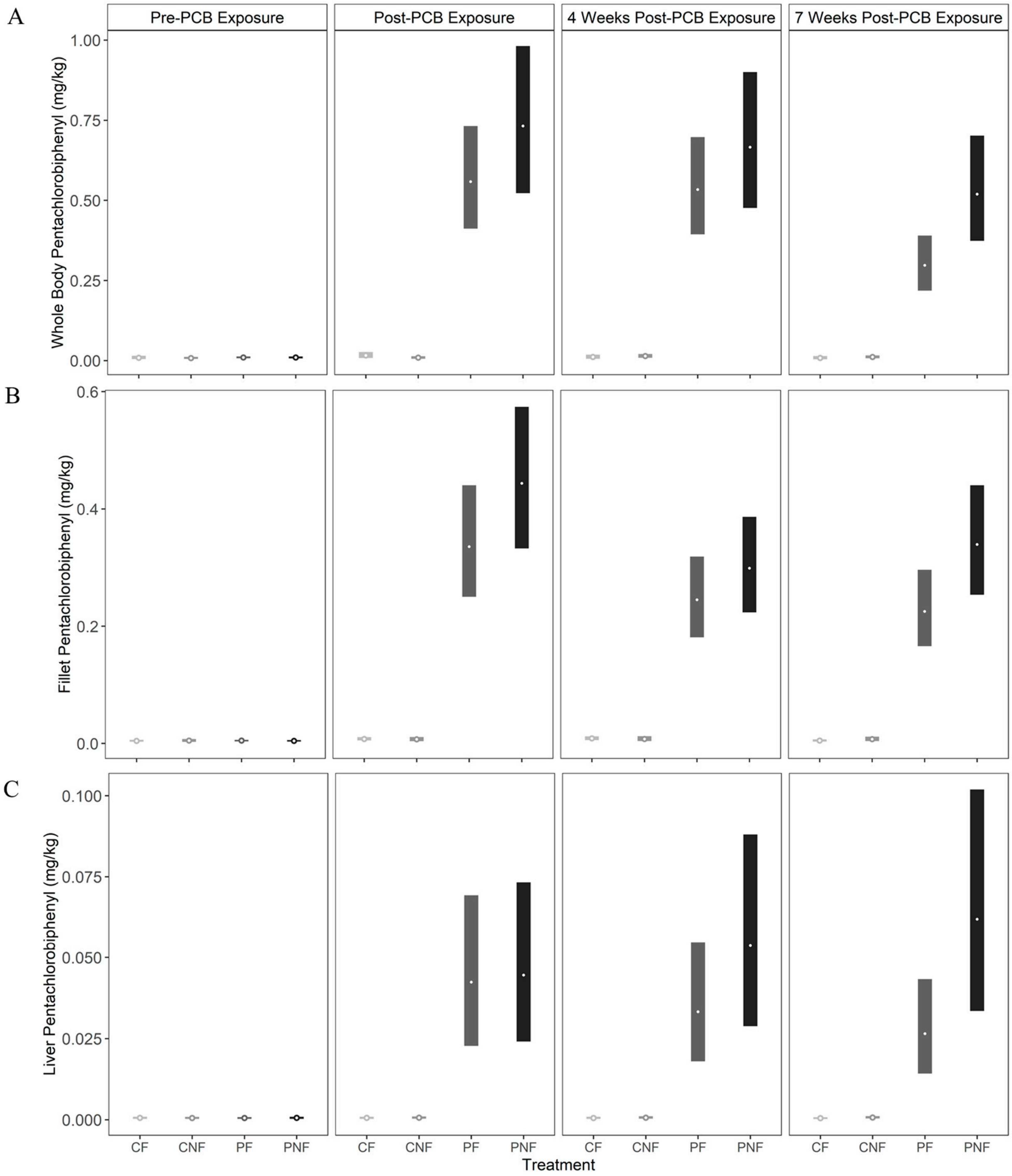

Figure S6: Posterior mean (open circles) and 90% credible intervals (bars) for pentachlorobiphenyl concentrations in w whole body (A), fillet (B), and liver (C) tissues for channel catfish in each of four treatments (CF = control and fed, CNF = control not fed, PF = PCB exposed and fed, PNF = PCB exposed and not fed). PCB concentrations were measured before PCB exposure, after 11 weeks of PCB exposure, and four and seven weeks after PCB exposure during which fish in the CNF and PNF treatments were also food deprived

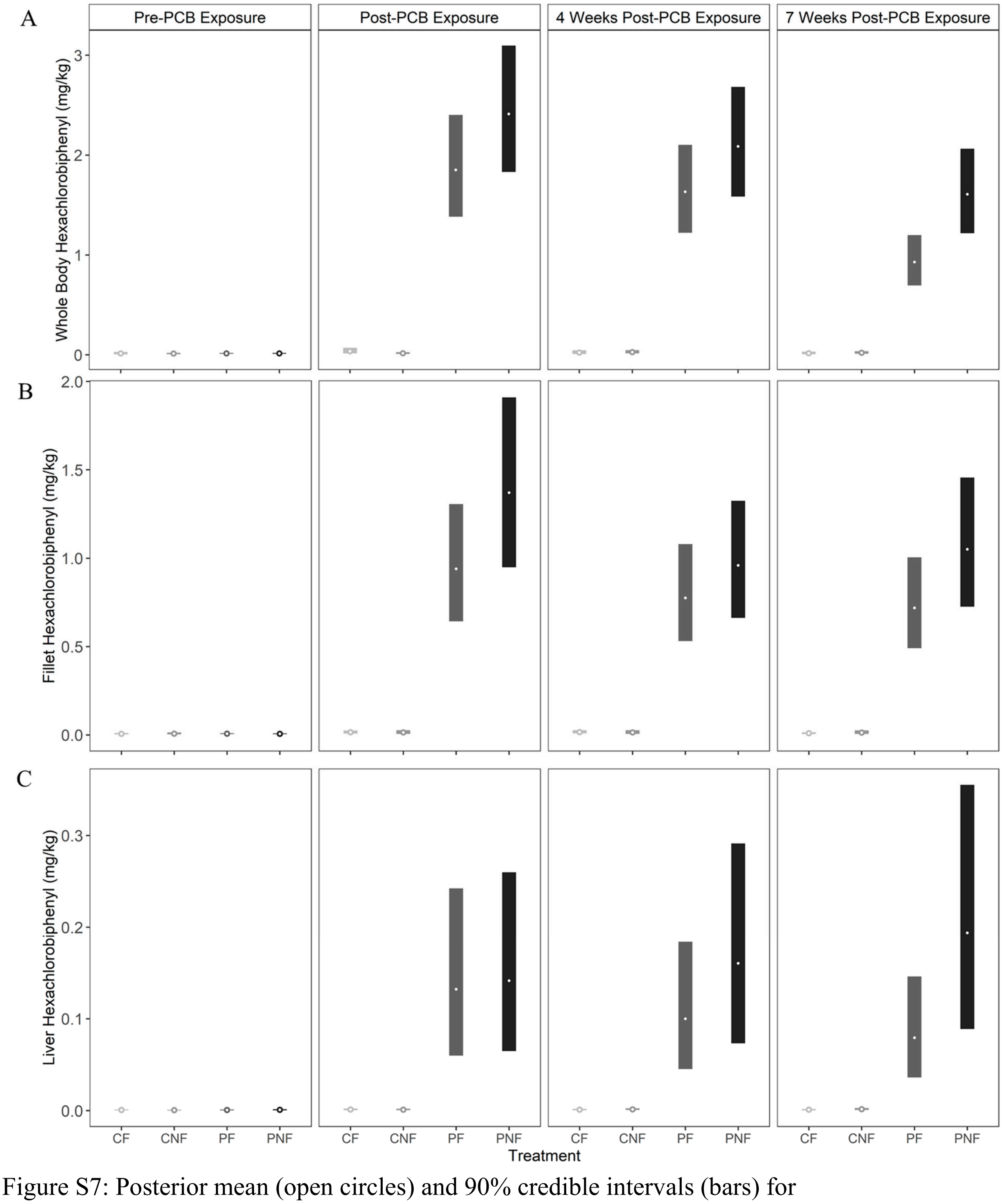

Figure S7: Posterior mean (open circles) and 90% credible intervals (bars) for hexachlorobiphenyl concentrations in whole body (A), fillet (B), and liver (C) tissues for channel catfish in each of four treatments (CF = control and fed, CNF = control not fed, PF = PCB exposed and fed, PNF = PCB exposed and not fed). PCB concentrations were measured before PCB exposure, after 11 weeks of PCB exposure, and four and seven weeks after PCB exposure during which fish in the CNF and PNF treatments were also food deprived.

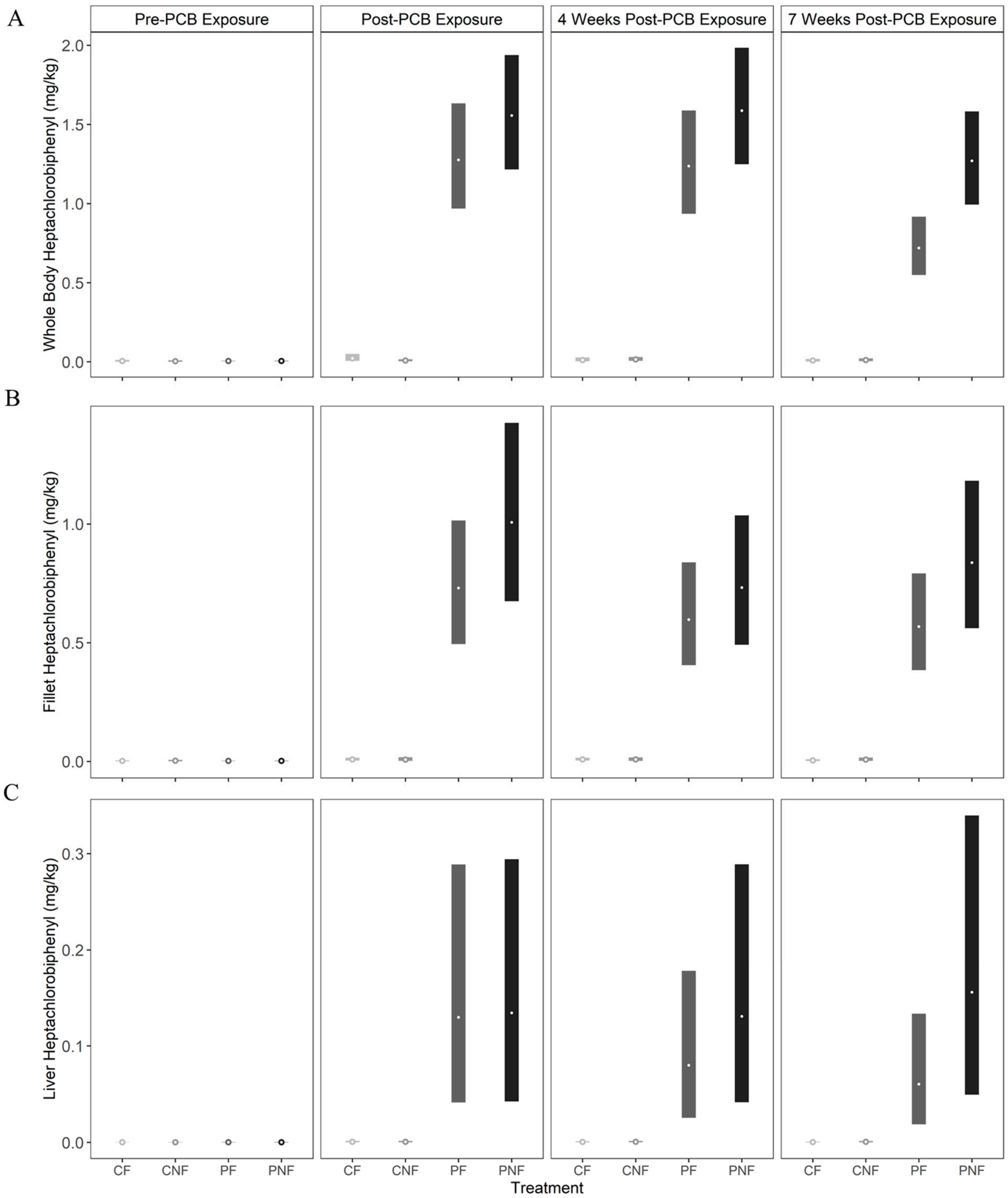

Figure S8: Posterior mean (open circles) and 90% credible intervals (bars) for heptachlorobiphenyl concentrations in whole body (A), fillet (B), and liver (C) tissues for channel catfish in each of four treatments (CF = control and fed, CNF = control not fed, PF = PCB exposed and fed, PNF = PCB exposed and not fed). PCB concentrations were measured before PCB exposure, after 11 weeks of PCB exposure, and four and seven weeks after PCB exposure during which fish in the CNF and PNF treatments were also food deprived.

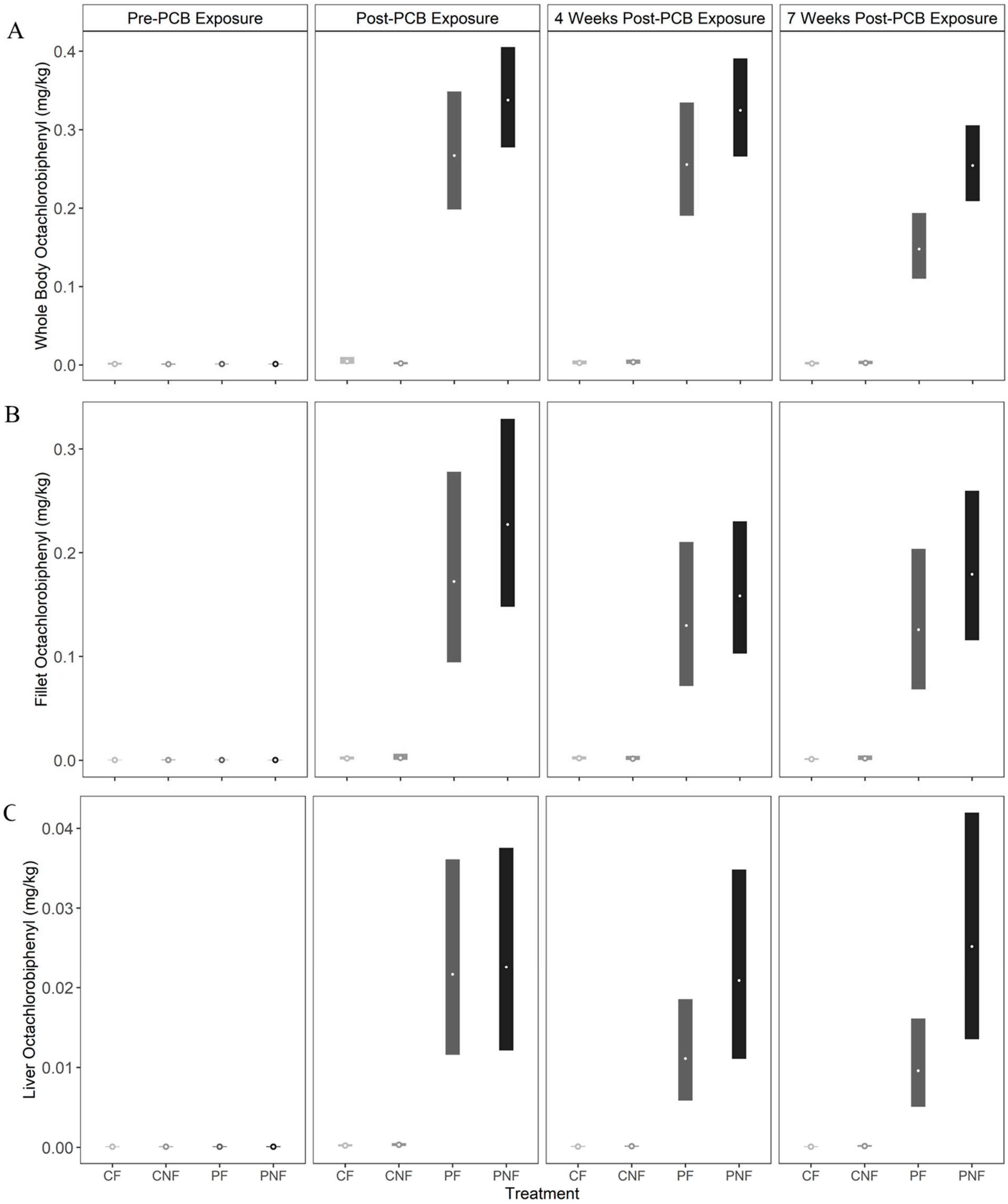

Figure S9: Posterior mean (open circles) and 90% credible intervals (bars) for octachlorobiphenyl concentrations in whole body (A), fillet (B), and liver (C) tissues for channel catfish in each of four treatments (CF = control and fed, CNF = control not fed, PF = PCB exposed and fed, PNF = PCB exposed and not fed). PCB concentrations were measured before PCB exposure, after 11 weeks of PCB exposure, and four and seven weeks after PCB exposure during which fish in the CNF and PNF treatments were also food deprived.

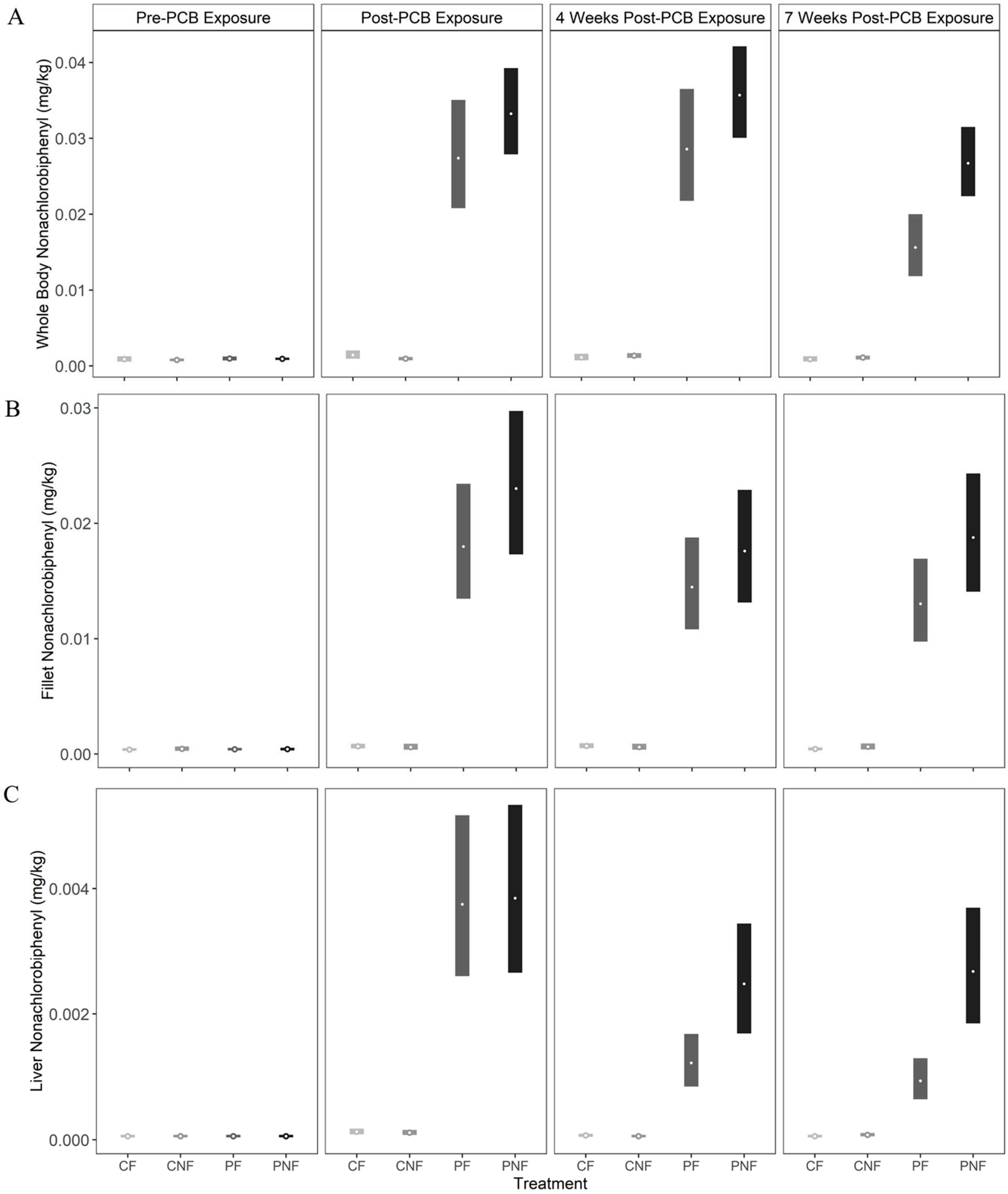

Figure S10: Posterior mean (open circles) and 90% credible intervals (bars) for nonachlorobiphenyl concentrations in whole body (A), fillet (B), and liver (C) tissues for channel catfish in each of four treatments (CF = control and fed, CNF = control not fed, PF = PCB exposed and fed, PNF = PCB exposed and not fed). PCB concentrations were measured before PCB exposure, after 11 weeks of PCB exposure, and four and seven weeks after PCB exposure during which fish in the CNF and PNF treatments were also food deprived.

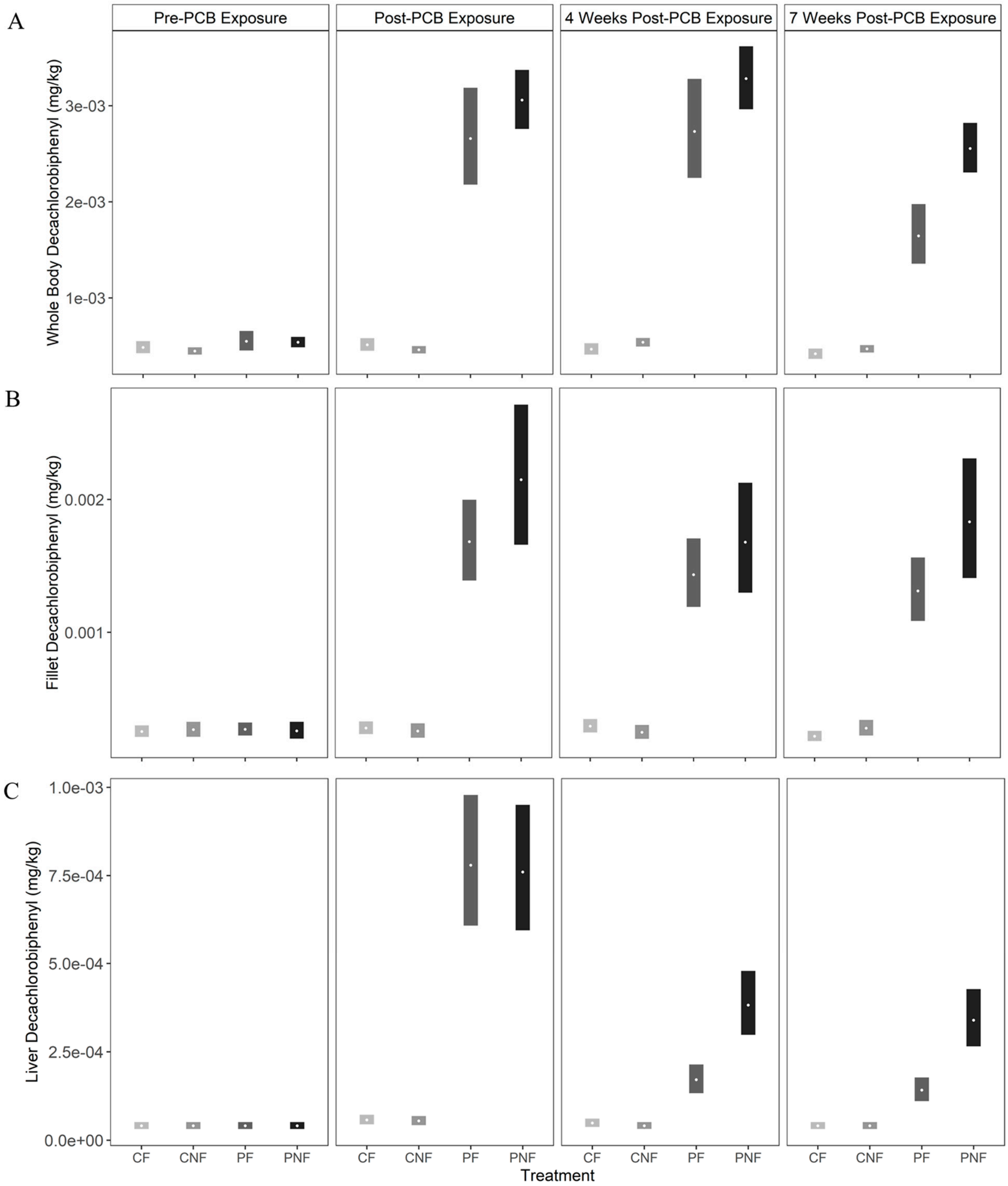

Figure S11: Posterior mean (open circles) and 90% credible intervals (bars) for decachlorobiphenyl concentrations in whole body (A), fillet (B), and liver (C) tissues for channel catfish in each of four treatments (CF = control and fed, CNF = control not fed, PF = PCB exposed and fed, PNF = PCB exposed and not fed). PCB concentrations were measured before PCB exposure, after 11 weeks of PCB exposure, and four and seven weeks after PCB exposure during which fish in the CNF and PNF treatments were also food deprived.
